# Function, Detection and Alteration of Acylcarnitine Metabolism in Hepatocellular Carcinoma

**DOI:** 10.3390/metabo9020036

**Published:** 2019-02-21

**Authors:** Shangfu Li, Dan Gao, Yuyang Jiang

**Affiliations:** 1 State Key Laboratory of Chemical Oncogenomics, Graduate School at Shenzhen, Tsinghua University, Shenzhen 518055, China; li.shangfu@sz.tsinghua.edu.cn (S.L.); jiangyy@sz.tsinghua.edu.cn (Y.J.); 2 National & Local United Engineering Lab for Personalized Anti-tumour Drugs, Graduate School at Shenzhen, Tsinghua University, Shenzhen 518055, China; 3 Key Laboratory of Metabolomics at Shenzhen, Shenzhen 518055, China; 4 School of Pharmaceutical Sciences, Tsinghua University, Beijing 100084, China

**Keywords:** acylcarnitines, hepatocellular carcinoma, metabolite profiling, metabolomics

## Abstract

Acylcarnitines play an essential role in regulating the balance of intracellular sugar and lipid metabolism. They serve as carriers to transport activated long-chain fatty acids into mitochondria for β-oxidation as a major source of energy for cell activities. The liver is the most important organ for endogenous carnitine synthesis and metabolism. Hepatocellular carcinoma (HCC), a primary malignancy of the live with poor prognosis, may strongly influence the level of acylcarnitines. In this paper, the function, detection and alteration of acylcarnitine metabolism in HCC were briefly reviewed. An overview was provided to introduce the metabolic roles of acylcarnitines involved in fatty acid β-oxidation. Then different analytical platforms and methodologies were also briefly summarised. The relationship between HCC and acylcarnitine metabolism was described. Many of the studies reported that short, medium and long-chain acylcarnitines were altered in HCC patients. These findings presented current evidence in support of acylcarnitines as new candidate biomarkers for studies on the pathogenesis and development of HCC. Finally we discussed the challenges and perspectives of exploiting acylcarnitine metabolism and its related metabolic pathways as a target for HCC diagnosis and prognosis.

## 1. Introduction

Hepatocellular carcinoma (HCC) is the most common type of primary liver cancer. This intra-abdominal malignant tumours accounted for 90% of all cases of primary liver cancer [1]. HCC ranks as the second leading cause of cancer-related mortality in the world [2]. It has a very poor prognosis of malignant tumours, with prognosis less than 5% [3]. The main pathogenic factors of HCC are viruses, bacteria, alcohol, therapeutic drugs, and harmful substances [4]. Its occurrence is long-term, dynamic, and multi-stage with the complex regulation of multiple genes and factors [5]. Chronic liver damage and inflammation caused by chronic hepatitis B virus or hepatitis C virus (HBV, HCV) infections account for the majority of HCC cases [6]. The persistent inflammatory environment may promote simple hepatic steatosis to fibrosis, cirrhosis (CIR) and, ultimately, HCC [2,7]. Additionally, in the last 20 years, the rising rates of alcoholic liver disease, non-alcoholic fatty liver disease (NAFLD), and non-alcoholic steatohepatitis (NASH) increased the risk of HCC development in patients with viral hepatitis [8]. In fact, these liver metabolic disorders, including type II diabetes, obesity, and metabolic syndrome, are becoming emerging risk factors for the rapidly increasing incidence of HCC [9]. It has been reported that 4% to 27% of patients with NASH and CIR may have HCC [10]. However, the oncogenic mechanisms of these new metabolic risk factors that promote HCC are only beginning to be characterized [11]. In order to improve the early diagnosis of HCC and the prognosis of patients, investigation of the pathogenesis of HCC and exploration of high-sensitivity, high-specificity biomarkers are the hotspots for HCC research. The development of current research techniques provides a great deal of convenience to investigate HCC-related biomarkers [12,13]. In particular, the omics technologies, such as genomics, proteomics, and metabolomics, have greatly accelerated the progress in HCC research with its high-throughput technology advantages [14,15]. The investigations provide many sensitive and specific markers for early and accurate diagnosis of HCC [16].

Since the liver is an important organ of substance and energy metabolism, liver lesions, especially carcinogenesis, can strongly affect its metabolic process [17]. Therefore, quantitative and qualitative analysis of metabolic alteration in HCC samples can monitor the fluctuation of specified metabolic pathways, thus obtaining some important information for the diagnosis and pathogenesis studies of HCC [18,19]. These are the currently booming research scopes of metabolomics in recent years [20,21]. At present, targeted and non-targeted metabolomics studies on HCC have been widely reported [22,23,24]. However, due to the large variety of metabolites, there is currently no single prospecting technique that can fully cover all metabolites [25]. Generally, only some of the metabolites of interest can be detected by quantitative or qualitative methods or a mix of both. In this article, we do not attempt to summarize the changes of all metabolites in HCC as well, but rather focus on the acylcarnitines, which are a large class of substances closely related to HCC metabolism.

## 2. Function of Acylcarnitines in Cellular Metabolism

**Figure 1:**
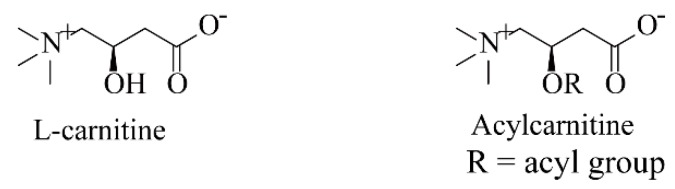
The structure of l-carnitine and acylcarnitines.

Acylcarnitines are esters of l-carnitine and fatty acids (Figure 1). They are a large class of metabolites that are members of the non-protein amino acid family. According to the Human Metabolome Database, there may be more than 1200 fatty acids in the human body [26,27]. Therefore, it is inferred that the number of acylcarnitines that may be formed with these fatty acids is very considerable. Similar to fatty acids, acylcarnitines are also differed by length of the acyl groups, often categorized as short, medium and long-chain acylcarnitines (simply marked as SCACs, MCACs and LCACs). Acylcarnitines are zwitterionic compounds, containing a carboxyl group and a quaternary ammonium group in the molecule (Figure 1). 

**Figure 2:**
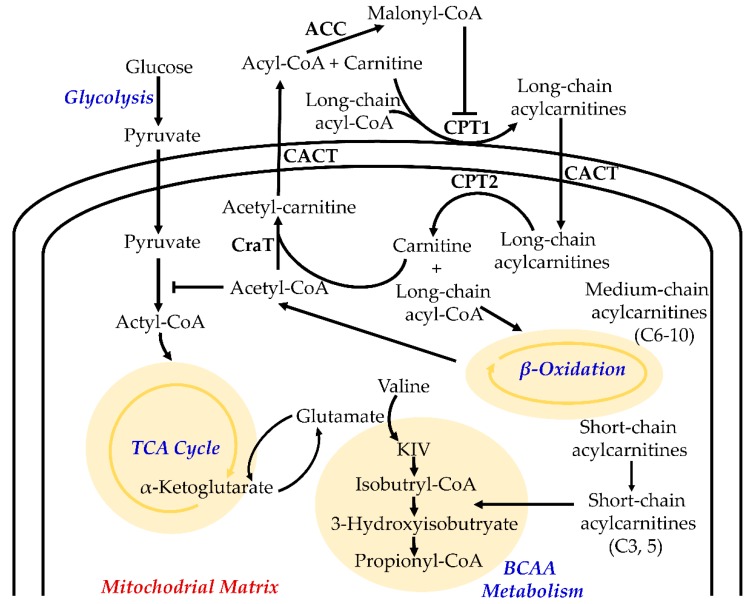
Overview of acylcarnitine-related cellular metabolism. For simplicity, not all intermediates and reversible processes are shown. Abbreviations: CPT1, carnitine-palmitoyl-transferase 1; CPT2, carnitine-palmitoyl-transferase 2; CACT, carnitine-acylcarnitine-translocase; CoA, coenzyme A; LACS, long-chain acyl-CoA synthetase; BCAA, branched-chain amino acid.

The large number and special structure make acylcarnitines play an important role in cell physiological activities and become a key substance for cell metabolism [28]. The main function of acylcarnitines is involved in long-chain fatty acids (LCFAs) β-oxidation (Figure 2). They serve as carriers to transport activated LCFAs into mitochondria for subsequent β-oxidation to provide energy for cell activities [29]. The enzymes that regulate these processes are mainly long-chain acyl-coenzyme A synthetase (LACS), carnitine/acylcarnitine translocase (CACT), carnitine palmitoyl-transferase 1 and 2 (CPT1 and CPT2) [30]. The LCFAs are activated by linking to coenzyme A (CoA) via LACS, forming long-chain acyl-CoAs. The intermediaries are converted into LCACs catalysed by CPT1 which is located on the outer mitochondrial membrane [31]. Under catalysis of CACT, the LCACs are imported through the mitochondrial membranes into the mitochondrial matrix [32]. Then they are converted back to the corresponding long-chain acyl-CoAs in the presence of CPT2 for β-oxidation [33]. The end products, acetyl-CoAs, are converted to acetylcarnitines by carnitine O-acetyltransferase (CrAT). Finally, acetylcarnitines are exported from mitochondrion to cytoplasm by CACT [34]. The activities of the involved enzymes can be evaluated by ratios of LCACs/SCACs. For example, the activity of CPT1 can be estimated by (carnitine C16 + carnitine C18)/carnitine. Similarly, the changes of CPT2 can be estimated by (carnitine C16 + carnitine C18:1)/carnitine C2, long-chain Acyl-CoA dehydrogenase by carnitine C16/carnitine C8, and β-oxidation of even-carbon fatty acids by carnitine C2/carnitine [35].

The metabolism of acylcarnitines is a key factor regulating the balance of intracellular sugar and lipid metabolism [36]. Acylcarnitine metabolism is involved in the metabolism of branched-chain amino acids [37]. They are also involved in maintaining the homeostasis of the mitochondrial acyl-CoA/CoA ratio. When the glucagon/insulin ratio is lowered, they stimulate the activity of pyruvate dehydrogenase to enhance the oxidation of pyruvate and enhance the aerobic oxidation of glucose [38]. Acetylcarnitine can be converted into malonyl-CoA in the cytosol to inhibit the activity of CPT1 and reduce the oxidation of fatty acids, which results in eliminating the adverse reactions caused by the accumulation of acyl-CoA metabolic intermediates in the mitochondria [39]. Acylcarnitines are also involved in other physiological processes such as peroxidation of fatty acids, and production of ketone bodies [37]. Therefore, the metabolism of acylcarnitines is not only related to the transport of fatty acids, but also plays a key role in regulating the balance of intracellular sugar and lipid metabolism (Figure 2) [36].

Acylcarnitines are closely related to many metabolic diseases [40]. Abnormal expression of enzymes involved in the metabolism of acylcarnitines may result in accumulation of acyl-CoA with a specific chain length [41]. These substances, if not removed by conversion to acylcarnitines, may have toxic effects on cells [42,43]. Since the levels of plasma acylcarnitines reflect the composition of the acylcarnitine pool within the cytoplasm, they are considered to be markers indicating the balance between acyl-CoA and acylcarnitine species [44]. Studies have shown that in organic acidemia, the content of acylcarnitines varies with the accumulation of organic acids. Therefore, acylcarnitines are clinically important parameters for organic acidemia diagnosis [45]. Acylcarnitines are also key indicators for screening genetic abnormalities in neonates [46]. In addition, changes of blood acylcarnitines also have significant correlation with type I diabetes and type II diabetes [47]. Mitochondrial fatty acid oxidation (FAO) disorders caused by gene mutations can lead to hereditary carnitine metabolism syndrome [48]. Secondary carnitine deficiency may be triggered by back of nutrition, absorption of gastrointestinal function, carnitine loss from blood and peritoneal dialysis [48]. Significant changes in acylcarnitine metabolism can also be observed in diseases such as coronary artery disease [49], heart failure [50], and dementia [51]. In cancer cells, acylcarnitine metabolism has been considered as a gridlock to finely trigger the metabolic flexibility of cancer cells on the basis of its fundamental role in tuning the switch between the glucose and fatty acid metabolism [34]. Metabolic reprogramming of cancer cells regulates the levels of acylcarnitines with varying chain lengths [52,53]. They intercalate acylcarnitines with other key metabolic pathways, factors and metabolites to create a balance between the production and consumption of energy and the synthesis of metabolic intermediates to meet rapid proliferation requirements [54,55]. For example, in prostate cancer cells (PCCs), carnitine cycle was a primary regulator of adaptive metabolic reprogramming, which was modulated by microRNAs (miRNAs) to deregulate mitochondrial fatty acid oxidation [56]. Results from the urine of kidney cancer patients and mouse models showed that urinary acylcarnitines are increased in a grade-dependent fashion. These compounds are likely emanating from the tumour tissue itself and have both cytotoxicity and immune modulatory properties which could be beneficial to the tumour in terms of growth and survival in situ [57]. Mass spectrometry images analysis found that in a human breast tumour xenograft model, two acylcarnitines, palmitoylcarnitine, and stearoylcarnitine, displayed the high percentage of overlap with hypoxic tumour regions, suggesting blockage of the β-oxidation process of fatty acids inside mitochondria [58]. In view of the importance of acylcarnitines in a variety of diseases, they are likely to be good biomarkers for clinical diagnosis. Therefore, studies on the function of acylcarnitines may help to deepen understanding of the disease mechanism, and it may also promote the development of disease diagnosis and treatment technology.

## 3. Detection of Acylcarnitines in Biological Samples

However, the difficulties encountered in the detection of acylcarnitines limit the study of their functions. The challenge for acylcarnitines detection is mostly attributed to the complex components of biological samples and the structural diversity of acylcarnitines caused by various acyl groups [37,59,60]. (1) The composition of biological samples is very complex. Matrix components will greatly interfere with the detection of acylcarnitines; (2) According to the different acyl groups attached, the acylcarnitines have a very long polarity span, covering the polarity from the polar SCACs to low-polar LCACs. SCACs have strong hydrophilicity due to the presence of quaternary ammonium groups and are difficult to retain on reversed-phase columns; (3) Due to a wide variety of species of fatty acids, the SCACs, MCACs and LCACs formed by the fatty acids constitute a large congener family of members, and the properties of some isomers are very close, leading to difficulty in chromatographic separation; (4) The limited number of commercial acylcarnitine standards affects the accurate identification of the specific structural composition of acyl groups. Due to the existence of these problems, the current detection methods can only focus on a few acylcarnitines that contain commercial standards, and the information of other acylcarnitines is still missed.

Biological samples usually contain macromolecules, such as proteins and nucleic acids, as well as small molecules such as phospholipids, amino acids, sugars, and inorganic salts. Therefore, the matrix effect caused by these ingredients cannot be ignored in the detection of acylcarnitines. Although there was a report that urinary acylcarnitines could be detected directly after the urine samples were subjected to simple centrifugation [61], the strong matrix effect still affected the sensitivity. Therefore, in order to efficiently detect acylcarnitines, appropriate sample preparation methods are necessary. The easiest way to handle the biological samples is the liquid–liquid extraction (LLE) method. Due to its convenient operation and low cost, it has been extensively used for sample preparation [62]. The usual procedure includes protein precipitation, centrifugation, and nitrogen drying [63]. The organic solvents used for deproteinization often are methanol (MeOH) or acetonitrile (ACN). Studies have shown that the choice of organic solvents has a great impact on the recovery of the methods, because acetonitrile itself is not a good solvent for all the acylcarnitine species [64]. Therefore, it is common to use MeOH or a mixture of MeOH and ACN for LLE [65,66,67]. In addition, using ACN containing 0.3% formic acid could also improve the extraction recovery, which was comparable to those approaches using MeOH as solvent [66]. The limitation of LLE method is that, it requires to use potentially toxic organic solvents and high volume samples, and its sampling rate is low. In response to these problems, some new methods have been developed for the extraction of acylcarnitines. For example, using a continuous-flow microelectroextraction flow cell, acylcarnitines could be extracted from a large volume urine sample into a micro volume of stagnant acceptor phase under the electric field enhanced extraction [68]. The detection limit of the method was as low as 0.3–2 nM, which was appropriate for the detection of low concentration metabolites.

Solid-phase extraction (SPE) is another widely used method for the extraction of acylcarnitines. Its advantages are low cost, good selectivity, small solvent consumption, small sample amounts and high recovery [69]. Additionally, its disadvantages are long sample preparation times and multistep procedures. Despite these shortcomings, SPE is still extremely applied for the enrichment and isolation of acylcarnitines. For example, after purifying human urine using polymeric and weak cationic exchange cartridges, the matrix effect was significantly reduced as the urinary carnitine was analysed by UPLC-MS/MS [70]. An online SPE with an Oasis mixed-mode cation exchange (MCX) trapping column combined with LC-MS analysis was a rapid sample work-up method for the quantification of acylcarnitines with different polarity. The method required low sample consumption [71]. The sample preparation was more simplistic, and LLOQ was significantly lower than previously reported methods [72,73] after treating samples with semi-automatic microextraction by packed C2 of M1 (C8 + SCX) phase as a sorbent [74]. It was also reported that using a mixed-mode reversed-phase/strong cation-exchange 96-well SPE plate could achieve selective and accurate quantitation of C5 acylcarnitine in patients with isovaleric acidemia (IVA) by UHPLC–MS/MS [75]. With the same SPE plate isolation, 65 acylcarnitines were separated [76]. Although MCX SPE cartridges have been widely used in the extraction of acylcarnitines, it must be noted that the sulfo group on the packing could catalyse the carboxylic acid groups of about 40% dicarboxylic acylcarnitines reacting with MeOH in the elution solvent to form methylation products [77,78]. Therefore, Li et al. suggested that using ACN (containing 5% NH_4_OH) instead of MeOH as elution may avoid the methylation problems [79].

The as prepared samples can be directly used for analysis [74,80], or analysed after derivatization. The derivatization procedure may introduce a chromophoric group to the targeted analytes, then it could be possibly detected by fluorescence or ultraviolet detector [81,82]. The derivatization procedure was also used to label water-soluble acylcarnitines to improve their retention on reversed-phase columns [83,84]. For example, the strategies have been used to detect l-carnitine and its chiral isomers d-carnitine [60]. And just recently, an isotope-labelling strategy with 3-nitrophenylhydrazine as derivatization reagents was employed for LC-MS-based quantitation of acylcarnitines in dried blood spots with good linearity, high precision and high accuracy [85]. One important aspect to note is that the application of derivatization methods requires systematically methodological evaluation of the chemical stabilities of acylcarnitines under various reaction conditions before they are used in the practical sample analysis. Since it has been confirmed that anhydrous n-butanol/HCl-based method, which was based on the acid-catalysed esterification and the most popular derivatization approach for acylcarnitines analysis at early stages [86], may cause part of the acylcarnitines hydrolysed and result in inaccuracies measurement from the hydrolysis of acylcarnitines [87]. 

Due to the complex composition of biological samples, proper separation means are beneficial for acylcarnitine analysis to obtain the maximum detection efficiency. As in the earlier study, chromatography separation coupled with fluorescence or UV detectors were commonly used methods [60,82]. There are also a small number of studies used GC-MS [88]. In recent years, LC-MS has become the most popular techniques for acylcarnitine detection [89,90,91,92,93]. Reversed-phase liquid chromatography (RPLC), hydrophilic interaction liquid chromatography (HILIC), ion chromatography, and capillary electrophoresis are different options for acylcarnitine separation. Due to their excellent separation ability and high sensitivity, dozens of acylcarnitines could be analysed simultaneously [94,95,96,97]. By using highly-selective scanning modes, such as selected reaction monitoring (SRM), multiple reaction monitoring (MRM) and parallel reaction monitoring (PRM), up to hundreds of acylcarnitines could be identified in plasma, urine and tissue samples [37,59,79]. These results provide significant reference value to annotation of acylcarnitines in biological samples. However, in these researches, especially for qualitative analysis, the identification of acylcarnitines relied primarily on accurate values of mass to charge ratio (*m/z*) and corresponding characteristic fragment ions obtained from high resolution mass spectrometry. Nevertheless, the majority of detected acylcarnitine structures cannot be actually verified because of not enough commercial standards for the diverse acylcarnitines. In some cases, such as the discovery of potential acylcarnitine biomarkers for clinical application, standards are still needed to be synthesized for their structures confirmation [79]. Although some of acylcarnitines can be obtained by conjugating the corresponding carboxyl compounds with l-carnitine, only a small fraction of currently known acylcarnitines can be synthesized. This is because the carboxyl compounds are also diverse and lack of sufficient standards. The un-authenticated acylcarnitines in biological samples limited the evaluation of these existing methods [59]. Therefore, the development of new approaches to high efficient and accurately identify acylcarnitines is still in urgent need. 

## 4. Alteration of Acylcarnitine Metabolism in HCC

The liver is the most active organ for endogenous carnitine synthesis and metabolism [91,98]. Therefore, suffering from diseases may cause the liver to have a strong effect on the levels of acylcarnitines [99]. At different stages of liver disease, hepatocytes are stimulated by different risk factors, and the demand for glucose and lipids is not the same [100,101]. As a result, the disorder of acylcarnitine metabolism is also related to the stage of liver disease. The general rule is that as the condition worsens, the metabolic disturbance of acylcarnitines becomes more pronounced. Some research suggested that in non-alcoholic fatty liver disease (NAFLD) patients, the level of butyrylcarnitine was significantly elevated. When the disease progressed to more severe NASH, there was a significant increase in free carnitine, propionylcarnitine, butyrylcarnitine, and 2-methylbutyrylcarnitine [102]. In patients with liver fibrosis and CIR, both C16:1-acylcarnitine and C18:1-acylcarnitine have an increasing tendency, indicating reduced β-oxidation levels of these two fatty acids [103]. The changes of acylcarnitines caused by different pathogenic factors are also different. For example, serum LCAC levels in patients with CIR caused by viral hepatitis (HBV and HCV) showed an increasing trend, but in patients with CIR caused by alcohol consumption, both LCACs and SCACs were upward trend [35,104]. 

There are also many reports on the changes of acylcarnitines in HCC. Compared with human HCC clinical samples, cell and animal models are relatively easy to obtain and can perform knockout, silencing, high expression and other operations on genes of interest and, thus, are often used to study the disease mechanism of HCC. For example, Cheng et al. established SK-Hep1 cells underexpressing G6PD (Sk-Gi) to study the effect of a pharmacological dose of dehydroepiandrosterone on cellular metabolism. Compared with control cells (Sk-Sc), consumption of carnitine and its acyl derivatives was observed, suggesting the decline in fatty acid catabolism and mitochondrial malfunction and reduction in cellular ATP content [105]. Levels of acylcarnitines also enhanced the self-renewal of HCC cells. It was reported that Dih10 cells with CPT2 knockdown led to their resistance to lipotoxicity induced by the lipid-rich cellular environment via inhibiting the Src-mediated JNK activation. Simultaneously, by stimulating STAT 3, oleoylcarnitine may promote sphere formation in Dih10 cells [54]. In hepatitis B surface antigen (HBsAg) transgenic mouse model that mimics HBV carriers with and without AFB1 treatment, acylcarnitine concentration increased with increase in tumour growth in all HCC mouse models, indicating elevated metabolic activity and increased cell turnover. The results were consistent with a pilot study using human serum from HCC patients [106]. In addition to endogenous acylcarnitines, externally added carnitine may also affect HCC progression. It was reported that l-carnitine increased hepatic expression of genes related to long-chain fatty acid transport, mitochondrial β-oxidation, and antioxidant enzymes following suppression of hepatic oxidative stress markers and inflammatory cytokines in NASH, and mice treated with l-carnitine developed fewer liver tumours [107]. By using a non-targeted metabolomics method, Xu et al. analysed the diethylnitrosamine-induced rat HCC disease model. The level of palmityl-l-carnitine showed different trends at different ages. It decreased with age at the early stage. However, it increased significantly after 8 weeks between the two groups. The results of a stepwise histopathological progression indicated that the model was similar to human HCC, and it had potential practicality of HCC diagnosis at early stages [108]. This is also a rare report on the use of animal models to study the changes of acylcarnitines. The experimental results of cells and animals provide a good reference for the mechanism of HCC regulation of acylcarnitine metabolism.

However, because the pathogenesis of HCC is very complicated, cell culture or animal models cannot accurately simulate this process [109,110,111]. The results obtained from these two models still do not accurately reflect the regulation mechanism of HCC on acylcarnitines [105,108]. Therefore, most studies have focused on the analysis of clinical samples. Among them, blood and urine samples are dominant. Few reports are related to tissue samples because of the difficulty in sample collection. Since the development of HCC is closely related to CIR and hepatitis [112,113,114], there are also many literatures that compare these liver diseases together. It is hoped to uncover the relationship between hepatitis, CIR and HCC, and it is also hoped to discover some specific markers that can distinguish these diseases. For example, Du et al. detected 14 characteristic metabolites with significant differences in the homogenate of tissue samples obtained from HBV-related HCC patients. Five of these metabolites (beta-sitosterol, quinaldic acid, tetradecanal, oleamide, and arachidyl carnitine) were first discovered in HCC samples [115]. Differential acylcarnitines found between HCC and liver disease control groups were listed in Table 1. Some detailed examples are discussed below.

Xu et al. used the LC-MS combined with the random forest–recursive feature elimination method to compare the serum metabolic profiling of patients with chronic liver diseases (CLD) and HCC. The results demonstrated the accumulation of LCACs and the decline of free carnitine, MCACs and SCACs were associated with the severity of liver disease. A corresponding change was observed in the related enzyme activities. And HCC had less effect on the general changing extent of acylcarnitines than the non-malignant liver diseases. The authors speculated that this might be possible due to the special energy-expenditure mechanism of HCC cells [116]. The alteration of carnitine C16:1 and carnitine C18:1 was found to be consistent in another report, which was proposed by a mutual information-support vector machine-recursive feature elimination method to filter out noise and non-informative variables during data processing. The accumulation of the two LCACs demonstrated that compared with control, severe liver diseases (CIR and HCC) presented more notable implications of metabolic changes related to fatty acid β-oxidation. Moreover, HCC could be discriminated from CIR by SM (d18:0/22:2 (OH)), pimelylcarnitine and carnitine etc. [117]. The authors also used a pseudotargeted approach for further confirmation the changes of acylcarnitines. Serum metabolomic analysis of patients with HCC showed that the levels of MCACs (C8, C8:1, C10, and C10:1) reduced and LCACs (C18:1 and C18:2) levels were raised [118]. The study from Ong et al. also confirmed the similar difference between SCACs, MCACs and LCACs in HCC. In addition, they further verified that serum acetylcarnitine was a highly accurate biomarker for HCC diagnosis and progression, especially for AFP false-negative HCC patients [119]. However, the changes of LCACs appeared to be related to the type of hepatitis virus. Since it was reported that the level of octadecadienyl carnitine was higher in HBV-associated CIR group than in CHB and HBV-associated HCC groups [35]. 

Shariff et al. used a NMR system to analyse urine samples from hepatitis B surface antigen (HBsAg)-positive patients with HCC, HBsAg positive patients with CIR, and HBsAg negative healthy controls in Nigerian subjects. It was found that four metabolites, including creatinine, carnitine, creatine, and acetone, were strongly contributed to the grouping of the samples. The carnitine levels in the HCC group were meaningfully higher than in the healthy and CIR groups, reflecting that carnitine was excessive produced to meet the requirement of mitochondrial activity and rapid growth of cancer cells [120]. The method was then applied to detect HCV infected Egyptian patients with HCC. The metabolic profile presented similarity to that of Nigerian patients. It was firstly reported that metabolic alteration of HCC patients in two etiologically and ethnically distinct populations was similar, proposed that metabolic disorder caused by tumourogenesis did not rely on the two factors [121]. However, compared with the results of LC-MS analysis, the two studies also showed that except for carnitine, other acylcarnitines were difficult to be observed by NMR. The same results were also demonstrated in other studies [122]. By contrast, a LC-MS-based targeted and non-targeted study on the sera samples of 40 HCC and 49 CIR patients from Egypt detected more notably different metabolites. The results confirmed that the bile acid metabolites, LCACs and small peptide showed significant differences between the HCC and CIR control group. Of these remarkable metabolites, LCACs, oleoyl carnitine, palmitoyl carnitine, and linoelaidyl carnitine were down-regulated in HCC patients compared with CIR controls [123]. 

Combining multiple techniques to analyse disease samples can overcome the bias of a single method, improving the coverage of metabolite detection to obtain more comprehensive results. For example, Liu et al. reported on the use of NMR and LC-MS for global metabolomics analysis of serum of HCC cases [124]. GC-MS and LC-MS analyses were employed to investigate serum metabolic abnormalities in HBV-CIR and HCC patients [125,126]. Another report was used GC-MS and UPLC-MS/MS platforms to analyse the global serum metabolomes of HCC, hepatitis C CIR disease and healthy controls. The most significant altered metabolites included fatty acids, amino acids and acylcarnitines. Of these, SCACs and MCACs were highly overexpressed in HCC patients compared to disease controls, while LCACs trended downward [127]. To choose different stationary phases for sample separation was also an effective method to increase the detection rate of metabolites. For example, using both HILIC and RPLC to separate the urinary metabolites could found carnitine C10:1, carnitine C8:1, butylcarnitine, acetyl carnitine and cartinine, carnitine C9:1, carnitine C10:3, and carnitine C9:0 as potential biomarkers [128,129]. Zhang et al. sampled the HCC and CIR patients’ blood samples on filter paper and dried at room temperature, then extracted using organic solvent and concentrated for mass spectrometry analysis. Using the detected amino acids, acylcarnitines and some of their relevant ratios as the evaluation criteria, it was found that in this model, in view of their individual odds ratios, C5-OH/C0, C3/Met, and Val/Phe seemed to be the most important risk factors for HCC, while Thr, C3DC/C10, and C18:1 seemed to be the risk factors for CIR [130]. 

The value of the area under the curve of the receiver operating characteristic curve (ROC) is commonly used as indicators for evaluating specificity and sensitivity of biomarker. A study on the urine samples of HCC and CIR discovered that combination of butyrylcarnitine and hydantoin-5-propionic acid could differentiate the two diseases. The area of the two metabolites under ROC curve was 0.786 and 0.773, respectively [23]. By comparing principal metabolic alteration obtained from 50 HCC tissue samples and 298 chronic hepatitis and CIR serum samples, it was found that betaine plus propionylcarnitine was efficient for distinguishing HCC from the two types of liver diseases with a 0.982 AUC value of the ROC curve, which was much better than that of a-fetoprotein (AFP, 0.697). The combination was useful for the diagnosis of both AFP false-positive and false-negative HCC patients [24]. Another study showed that undecanoyl- l-carnitine, whose level was lower in HCC than in HBVs and NCs, in combination with α-fetoprotein could provide highly sensitive and specific for HCC diagnosis. The values of the area under the curve of the ROC curve was 0.92 [131]. This finding demonstrated that the combination of differential biomarkers presented good diagnostic potential to HCC.

Nielsen et al. established a genome-scale hepatocyte metabolic model and used system biology to analyse the metabolic changing of different HCV progressions. The levels of acylcarnitines were disturbed markedly in the dysplastic nodule and early HCC stages. This was related to the up-regulated genes, including BCAT1, PLOD3 and six other methyltransferase genes, which influenced carnitine biosynthesis and S-adenosylmethionine metabolism. Meanwhile, acyl-CoA consumption was regulated by GNPAT and BCAP31 upregulated expression. These genes could be used as potential targets for the therapy of liver disorders related to HCV [132]. 

Although the acylcarnitines with significant differences were not exactly the same in different reports due to the different samples, instruments and detection methods, the general rule of changes can still be in conclusion. It is certain that HCC can significantly regulate the metabolism of acylcarnitines. The levels of serum acylcarnitines in HCC patients showed specific patterns, mainly including increased levels of free carnitine, decreased levels of SCACs and MCACs, and increased levels of LCACs [116,118,133]. The main role of SCACs and MCACs is to remove organic acids from organelles such as mitochondria and excrete them to the urine and bile. The declining levels in serum indicated an increase in excretion rate of organelles or an obstacle of accumulation rate. Conversely, the promoted formation of LCACs in cells demonstrated increasing β-oxidation and producing more energy duo to the enhanced transport of LCFCs into the mitochondria [117]. However, it should be noted that there are low correlations between LCACs and SCACs/MCACs according to the results of acylcarnitine metabolic profiling from 80 pairs of HCC tissues and adjacent noncancerous tissues (ANTs) [135]. Among these significantly different acylcarnitines, some have been considered to have specificity of distinguish HCC from chronic hepatitis and CIR. It demonstrates the great potential of acylcarnitines as biomarkers for HCC diagnosis.

The mechanism of HCC-driven acylcarnitine changes has also been studied intensively. CPT1A and CPT2 are rate-limiting enzymes of LCFAs for β-oxidation [36,136]. Therefore, their expression is closely related to the changes of acylcarnitine levels. It was reported that in 66 post-operative liver tumour tissue from patients with resected HCCs, decreased expression of CPT1A was observed. And the expression changes appeared to correlate with risk factors for the prognosis of HCC patients, such as tumour size, histological grade, intrahepatic metastasis, and tumour–node–metastasis stage [137]. However, in another analysis based on the eighty pairs of HCC tissues and adjacent noncancerous tissues (ANTs), CPT1A expression was not significantly changed. These inconsistent findings suggest that the effect of CPT1A expression on the metabolism of acylcarnitines still requires further confirmation. In the later study, it was also found that downregulation of CPT2 was significantly associated with the presence of vascular invasion and poor tumour differentiation in HCC. And it caused low efficiency of the carnitine shuttle system, inducing the suppression of fatty acid β-oxidation in HCC. However, the downregulation of CPT2 could promote tumourigenesis, chemoresistance to cisplatin and lipogenesis [136]. Another independent study also identified CPT2 downregulation in HCC as a critical determinant in acylcarnitine accumulation. HCC cells presented resistance to lipotoxicity by the Src-mediated JNK inhibition after CPT2 was knocked out. In particular, oleoylcarnitine may act as an oncometabolite in hepatocarcinogenesis as it could promote HCC cell sphere formation by activating STAT3. Simultaneously, downregulation of CPT2 may mediate the metabolic reprogramming of HCC cells, which enables them to escape lipotoxicity and promotes hepatocarcinogenesis. These finding indicated that acylcarnitine accumulation was a surrogate marker of CPT2 downregulation [54]. These promising results offered mechanistic insights into acylcarnitine accumulation in HCC. As acylcarnitine metabolism is especially important for energy production in HCC, targeting this pathway is considered to be a potential strategy for cancer treatment.

## 5. Conclusions and Perspectives

Due to their special structure and function, the alteration of acylcarnitines in HCC has attracted significant attention. Certain acylcarnitines have been reported to present regular changes in HCC. These differential acylcarnitines have the potential to serve as biomarkers for HCC diagnosis. However, limited by the sensitivity of current detection techniques and the number of commercially available standards, only a small fraction of the known acylcarnitines could be accurately detected. The changes and function of these undetected acylcarnitines remain unknown in HCC. Therefore, it is urgent to develop new methods with high sensitivity and high selectivity to cover the detection of these metabolites as much as possible. Application of highly-selective sample preparation methods to enrich acylcarnitines and to reduce matrix interference may increase the probability of detection of low abundance acylcarnitines. In addition, the strategy of isotope labelling may be used for the relative quantification to overcome the problem of lack of standards. However, accurate structural identification and absolute quantification are still required in clinical detection. Therefore, obtaining the standards of acylcarnitines as many as possible is still the best way of approaching the problems.

In addition, the levels of acylcarnitines in HCC are affected by a number of factors, such as diet, renal dysfunction, biosynthesis rate, and other liver diseases. Therefore, in examining the regulation of HCC on acylcarnitines, the effects of these factors must also be carefully considered. In addition, acylcarnitine metabolism is an important node in the complex metabolic network of cells. Their levels are also affected by upstream and downstream changes in the metabolic pathways. To investigate the flux of acylcarnitines along the pathways may offer wonderful insight into the regulation mechanism of HCC on the acylcarnitine metabolism. The goals could be reached by accurate quantification of these metabolites using targeted metabolic profiling and metabolic flux analysis. With these comprehensive detection methods, some significantly differential acylcarnitines or their related metabolites may be discovered. They could be used as potential biomarkers for the subsequent study of HCC diagnosis or targets for drug development, which may supply a valuable reference for the pathogenesis and treatment investigation of HCC.

## Figures and Tables

**Table 1:** Differential acylcarnitines between HCC and liver disease control groups.

Reference	Sample	Platform	Main Findings
[23]	Urine 27 CIR 33 HCC 26 HC	LC-MS Non-targeted	HCC *vs*. CIR: MCACs and SCACs increased. CIR & HCC *vs*. HC: LCAC and MCAC decreased. HCC *vs*. CIR: carnitine C4:0 elevated
[24]	Tissues 50 HCT 50 DNT Serum 81 CH 78 CIR 139 HCC	LC-MS CE-MS Non-targeted Targeted	HCT *vs*. DNT: carnitine (C2/C0) upregulated, propionylcarnitine to carnitine (C3/C0) downregulated, SCAC decreased, LCAC increased HCC *vs*. CIR and CH: propionylcarnitine decreased, acetylcarnitine elevated
[35]	Sera 136 CHB 104 CIR 95 HCC	LC-MS Targeted	CIR and HCC *vs*. CHB: carnitine, 2 SCAC, and 4 MCAC decreased. CIR *vs*. CHB and HCC: AC C18:2 higher, AC C3-OH lower
[105]	SK-Hep1 cells underexpressing G6PD (Sk-Gi) and control cells (Sk-Sc) after dehydroepiandrosterone (DHEA) treatment	LC-MS Non-targeted	Carnitine and acyl derivatives declined in DHEA-treated Sk-Gi cells
[108]	Rat sera 52 HCC 28 HCHuman sera 262 HCC 76 CIR 74 HBV	LC-MS Non-targeted	HCC *vs*. HC (rat): palmityl-l-carnitine decreased (with the age of the animals), increased in week 8
[115]	HBV-related HCC tissue 10 CTT 10 ANT 10 DNT	LC-MSNon-targeted	DAT *vs*. CTT: arachidyl carnitine lower
[116]	Sera 30 HC 30 CHB 30 CIR 30 HCC	LC-MS Non-targeted	CHB *vs*. CIR *vs*. HCC: C16:1-CN increased (with severity of chronic liver diseases), MCAC and SCAC decreased (including C10-CN, C10:1-CN, C8-CN, and C6-CN) CIR and HCC *vs*. HC: C14-CN increased
[117]	Sera 30 CHB 30 CIR 30 HCC 30 HC	LC-MS Non-targeted	HCC *vs*. CHB: pimelylcarnitine and acetylcarnitine rise HCC *vs*. CIR: pimelylcarnitine and carnitine rise CIR and HCC *vs*. CHB and HC: C16:1-CN and C18:1-CN accumulated
[119]	Tissue 50 HCC (38 males and 12 females). Serum 18 HCC 20 CIR 20 HC	LC-MS Non-targeted	CTT *vs*. ANT: LCAC accumulated, MCAC and SCAC decreased. Acetylcarnitine reduced (gradually) HCC *vs*. CIR and HC: serum acetylcarnitine reduced
[120]	Urine 18 HCC 10 CIR 15 HC	NMR Non-targeted	HCC *vs*. CIR and HC: carnitine raised
[121]	Urine and serum 16 HCC 14 CIR 17 HC	NMR Non-targeted	HCC *vs*. CIR and HC: urinary carnitine elevated
[122]	Urine 42 HCC 47 CIR 7 HC	NMR Non-targeted	HCC *vs*. HC: carnitine increased
[123]	Sera 40 HCC 49 CIR	LC-MS Targeted Non-targeted	HCC *vs*. CIR: LCAC, oleoyl carnitine, palmitoyl carnitine, and linoelaidyl carnitine down-regulation
[124]	Serum 71 HCC 80 CIR 26 HC	NMR and LC-MS Non-targeted	HCC *vs*. CIR and HC: acylcarnitine increased, MCAC and LCAC decreased (including decanoyl-l-carnitine and palmitoylcarnitine) CIR and HCC *vs*. HC: carnitine decreased (progressive)
[125]	Serum 39 HC 49 HBV-CIR 51 HCC	LC-MS Non-targeted Targeted	HBV-CIR and HCC *vs*. HC: carnitine altered (stepwise)
[126]	Serum and urine 82 HCC 71 HC	LC-MS Non-targeted	HCC *vs*. HC: serum carnitine 1.36 times
[127]	Serum 30 HCC, 27 CIR 30 HC	LC-MS Non-targeted	HCC *vs*. CIR: acetylcarnitine, glutarylcarnitine and succinylcarnitine upregulated CIR *vs*. HC: MCAC overexpression HCC *vs*. CIR: LCAC downward
[128]	Urine 24 HC 21 HCC	LC-MS Non-targeted	HCC *vs*. HC: carnitine C8:1, carnitine C9:0, carnitine C9:1, carnitine C10:1 and carnitine C10:3 reduced, acetylcarnitine increased
[129]	Serum 24 HCC 25 CIR 25 HC	LC-MS Non-targeted	HCC and CIR *vs*. HC: carnitine, carnitine fragment and acetylcarnitine increased
[130]	Serum 92 male and 44 female CIR 41 male and 9 female HCC	LC-MS Targeted	HCC *vs*. CIR: decanoylcarnitine, decenoylcarnitine, propionylcarnitine/methionine, Methylmalonylcarnitine, 3-Hydroxy-isovalerylcarnitine/carnitine increased; octadecenoylcarnitine, malonylcarnitine/decanoylcarnitine, butyrylcarnitine/octanoylcarnitine decreased
[131]	Serum 267 HCC 48 HBV 272 HC	LC-MS Non-targeted	HCC *vs*. HC: SCAC and MCAC decreased, LCAC increased
[133]	22 HBV 6 HCV 38 HBV-associated HCC 31 HCV-associated HCC 31 HC	LC-MS Targeted Non-targeted	HCC *vs*. HC: C18:1-CN and C18:2-CN increased.
[134]	Serum 30 HC, 30 CHB, 30 CIR 30 HCC	LC-MS Non-targeted	CIR and HCC *vs*. HC and CHB: C16:1-CN and C16:0-CN elevated CHB, CIR and HCC *vs*. HC: C10-CN decreased, LCAC elevated, MCAC and SCAC exhibited the opposite trend

Abbreviation: Cirrhosis (CIR), hepatocellular carcinoma (HCC), healthy control (HC), hepatitis B virus (HBV), hepatitis C virus (HCV), chronic liver disease (CLD), chronic hepatitis B (CHB), chronic hepatitis (CH), adjacent noncancerous tissue (ANT), distal noncancerous tissue (DNT), central tumour tissue (CTT), hepatocellular carcinoma tissue (HCT), medium-chain acylcarnitine (MCAC), short-chain acylcarnitine (SCAC), and long-chain acylcarnitine (LCAC).
